# The Effects of Lentivirus-Mediated Gene Silencing of *RARβ* on the Stemness Capability of Non-Small Cell Lung Cancer

**DOI:** 10.7150/jca.50793

**Published:** 2021-04-19

**Authors:** Noor Hanis Abu Halim, Norashikin Zakaria, Kumitaa Theva Das, Juntang Lin, Moon Nian Lim, Kamal Shaik Fakiruddin, Badrul Hisham Yahaya

**Affiliations:** 1 Lung Stem Cell and Gene Therapy Group, Regenerative Medicine Cluster, Advanced Medical and Dental Institute (IPPT), Sains@Bertam, Universiti Sains Malaysia, Kepala Batas Penang, 13200, Malaysia.; 2 Infectomics Cluster, Advanced Medical and Dental Institute (IPPT), Sains@Bertam, Universiti Sains Malaysia, Kepala Batas Penang, 13200, Malaysia.; 3 Henan Joint International Research Laboratory of Stem Cell Medicine, Xinxiang Medical University (XXMU), Henan Province 453000, China.; 4 Stem Cell and Biotherapy Technology Research Centre of Henan Province, Xinxiang Medical University (XXMU), Henan Province 453000, China.; 5 Stem Cell Laboratory, Haematology Unit, Cancer Research Centre (CaRC), Institute for Medical Research (IMR), National Institute of Health, Setia Alam, 40170 Shah Alam, Selangor.

**Keywords:** non-small cell lung cancer, gene silencing, RAR1-beta, cancer stem cells

## Abstract

Retinoic acid receptor beta is a nuclear receptor protein that binds to retinoic acid (RA) to mediate cellular signalling in embryogenic morphogenesis, cell growth, and differentiation. However, the function of *RARβ* in cancer stem cells (CSCs) has yet to be determined. This study aimed to understand the role of *RARβ* in regulating cell growth and differentiation of lung cancer stem cells. Based on the clonogenic assay, spheroid assay, mRNA levels of stem cell transcription factors, and cell cycle being arrested at the G0/G1 phase, the suppression of *RARβ* resulted in significant inhibition of A549 parental cell growth. This finding was contradictory to the results seen in CSCs, where *RARβ* inhibition enhanced the cell growth of putative and non-putative CSCs. These results suggest that *RARβ* suppression may act as an essential regulator in A549 parental cells, but not in the CSCs population. The findings in this study demonstrated that the loss of *RARβ* promotes tumorigenicity in CSCs. Microarray analysis revealed that various cancer pathways were significantly activated following the suppression of *RARβ*. The changes seen might compensate for the loss of *RARβ* function, CSCs population's aggressiveness, which led to the CSCs population's aggressiveness. Thus, understanding the role of *RARβ* in regulating the stemness of CSCs may lead to targeted therapy for lung CSCs.

## Introduction

Lung carcinoma is the most common cause of cancer-related mortality worldwide. In Malaysia, at 10.2%, this is the third-highest cancer incidence after breast and colorectal cancer. Lung cancer, which is often diagnosed at an advanced stage, has a poor prognosis with a survival rate of 15% 1, causing it to be the leading cancer killer in both men and women 2. There are two main categories of lung tumours; small cell lung cancer (SCLC), which represents 20% of all lung cancers, and non-small cell lung cancer (NSCLC), representing 80% of total lung cancer incidence. NSCLC can be subdivided into three major histologic subtypes, including adenocarcinoma (AC), squamous cell carcinoma (SCC), and large cell carcinoma (LCC) 3.

Current treatments fail to eradicate cancer cells primarily due to tumour growth completely and spread driven by cancer stem cells (CSCs), minority cancer cells that exhibit similar characteristics as normal stem cells 4. The similarities of CSCs and normal stem cells lie in their proliferation, self-renewal, and differentiation, and their ability to initiate tumours - traits linked to therapeutic resistance 5. The existence of CSCs was first reported in acute myeloid leukaemia, where only a small portion of harvested leukemic cells could reproduce the same cancer in immune-deficient mice 6.

Three unique properties can identify CSCs; (1) it expresses a distinctive set of surface biomarkers, which allows it to be reproducible and have differential purification; (2) it has a selectively endowed tumorigenic capacity compared to other subsets; (3) it has the ability to recreate a repertoire of cancer cells of the parent tumour, thus displaying two of the functional characteristic of stem cells: self-renewal and differentiation 4. Several studies suggested that the putative CSCs population was identified based on specific surface markers' expression within solid malignancies, including EpCAM and CD166 7-10.

Epithelial cell adhesion molecule (EpCAM), also known as CD326 11, is a glycosylated, 30-40 kDa type I transmembrane glycoprotein that functions as an epithelial-specific cell-adhesion molecule 12,13. It is expressed in various human epithelial tissues, cancers, progenitor, and stem cells 13. CD166, another cell adhesion molecule, also known as ALCAM (activated leukocyte cell adhesion molecule), is associated with adenoma to carcinoma development 14. CD166 has been observed to be up-regulated in highly metastasising melanoma cell lines, suggesting a tumour migration role. These surface markers had been reported to be strongly expressed in carcinomas of various origins, including lung 15, colon and rectum 16, prostate 17, liver, and oesophagus 18, head and neck 19, and pancreas 20.

Other than CSCs, other factors also lead to cancer development. One of them is retinoid, a class of chemical compounds that regulates cell growth and differentiation of cells and apoptosis via retinoic acid cell signalling. Retinoid, an analogue of vitamin A, can reverse premalignant lesions, and prevent second primary tumour in some NSCLS patients 21. Retinoid suppresses carcinogenesis in various animal models for lung cancer, such as in rats 22. Retinoid requires its nuclear retinoid, retinoic acid receptors (RARs), and retinoid X receptors (RXRs) to mediate its function 23. Three distinct genes encode both RAR and RXR receptors; α, β, and γ (RARα, RARβ, RARγ, RXRα, RXRβ, and RXRγ gene). RARs and RXRs form heterodimers that bind to specific RA-responsive elements (RAREs) to regulate target gene expression. *RARβ,* which is the best characterised RA-responsive gene, is widely expressed in epithelial cells, has four alternative splice forms and the beta-2 form appears to possess tumour-antagonising activity. Studies on transgenic mice expressing antisense *RARβ*2 transcripts show lung tumours' development in contrast to non-transgenic control mice 24. Previous studies also reported that the *RARβ* gene was not expressed in several human cancer cell lines; H146, CALU-6, SK-MES1, H661, NCI-H23, and NCI-H125, suggesting a possible correlation between abnormal expression of the *RARβ* and lung cancer development 23; 25. However, a recent study reported that *RARβ* acts as a tumour suppressor and as a tumour promoter depending on the splicing variant expressed by the cells 25.

A previous study on *RARβ* showed that down-regulation of *RARβ* by antisense oligonucleotide resulted in down-regulation of several other genes with well-defined roles in carcinogenesis, including *BIRC5* 25. *BIRC5* gene (survivin) that encodes for BIRC5 protein, which is the smallest member of the inhibitor of apoptosis protein (IAP) 26, is strongly expressed in fetal tissues and typical human cancer cells 18. The ability of BIRC5 to stop apoptosis by inhibiting caspase-3 and caspase-7 leads to the assumption that this protein might play an essential role in tumorigenesis. This assumption was proved by previous studies that reported overexpression of this protein induces high proliferation levels in breast cancer MCF-7 cell line 27.

Other genes that were down-regulated resulting from the treatment of *RARβ* antisense oligonucleotide include cytochrome P450 family 24 (*CYP24A1*) and endothelin 1 (*EDN1*) 25. However, these genes were found to be upregulated following treatment with all-trans retinoic acid (ATRA), suggesting that the expression of these genes was regulated by retinoic acid 28. *CYP24A1* gene was believed to reduce the efficacy of certain anti-proliferative agents such as vitamin D and its analogues 29, and the *EDN1* gene that was known to act as a neoangiogenic and mitogenic factor in several cancers 30 are the best target of cancer therapy study.

Instead of *RARβ*, *ALDH1A1*, *BIRC5*, *EDN1*, and *CYP24A1* gene, prostaglandin-endoperoxide synthase 2 (*PTGS2*) as that is presumably downstream of *RARβ* 31 is responsible for many inflammatory processes. It is overexpressed in a variety of different tumours including the colon, pancreas, lung, and head and neck cancer. PTGS2 is an enzyme that plays a pivotal role in converting arachidonic acid to prostaglandins. Retinoids have prevented the induction of PTGS2 by mitogenesis and tumour promoter, thus this indicates that dysregulation of normal retinoid response may fail to downregulate PTGS2. Instead of the other six cancer-related genes, *IL1β* is also listed as a potential target of lung cancer study. A previous study reported that this gene is involved in *RARβ*2-mediated tumour suppression by enhancing the immune attack 32.

Therefore, characterising *RARβ* may help us better understand how retinoids regulate cell growth and differentiation, thus suppressing carcinogenesis. As the association of *RARβ* with CSCs is yet to be determined, this study aimed to understand the regulation of *RARβ* in CSCs maintenance and function. Understanding the role of *RARβ* in CSCs stemness, maintenance, and regulation might lead to targeted therapy for lung CSCs in the future.

## Materials and Methods

### Cell Culture

The human lung cancer cell line A549 (ATCC® CCL-185™) was purchased from American Type Culture Collection (ATCC, Manassas, VA, USA). A549 was cultured in ATCC-formulated RPMI-1640 medium supplemented with 10% foetal bovine serum (FBS) and 1% penicillin/streptomycin (Gibco, Thermo Fisher Scientific, MA, USA). Cells were cultured in a humidified incubator containing 5% CO_2_ at 37 °C and maintained in culture at confluency no greater than 90%. The culture medium was replaced every two to three days.

### Isolation of putative cancer stem cells

Putative CSCs were isolated based on the expression of CSC surface markers, CD166, and EpCAM, as previously described 7,33. Briefly, the cell suspension was incubated with antibody dilution buffer containing CD16/CD31 to block unspecific Fc interaction. Then the cells were labelled with PE-conjugated anti-CD166 (Clone: 3A4; Isotype: Mouse IgG1, κ) (BD Biosciences, San Jose, CA, USA) and FITC-conjugated anti-EpCAM (Clone: 158206; Isotype: Mouse IgG2B; Isotype: Mouse IgG1, κ) (R&D System, Minneapolis, MN, USA). The cells were incubated with the antibodies for 30 min on ice in the dark and then washed with PBS to remove unbound antibodies. The labelled cells were analysed, and cells population expressing CD166 and EpCAM marker (CD166^+^EpCAM^+^) and cells population that are negative for both markers (CD166^-^EpCAM^-^) were sorted out using FACSAria Fusion flow cytometry cells sorter (BD Bioscience). CD166^+^EpCAM^+^ and CD166^-^EpCAM^-^ cells subpopulations were cultured and expanded *in vitro* for subsequent experiments.

### Determination of Lentiviral Titer

Lentiviral stock carrying shRNA for human *RARβ* gene (NM_020143) and lentiviral vector carrying non-silencing control shRNA were purchased from DharmaconTM (Dharmacon Inc, Chicago, IL, USA). A transfer vector coding for shRNA against *RARβ* also contained TurboGFP, a green fluorescent protein (GFP) reporter used as an indicator for transfection efficiency. Different lentiviral dilutions were transduced into A549 cells to determine the transduction efficiency of the lentivirus. Twenty-four hours pre- transduction, 5×10^4^ cells were seeded per well in a 24-well plate. Serial dilution of the lentiviral particle was performed to obtain a five-fold dilution, and a final dilution of 390,625 X. Culture medium was removed from the cells and replaced with a serum-free medium. The diluted virus was then added to the cells and incubated at 37 ºC for 6 hours. Then, 1 ml of culture medium containing serum was added to the wells, and cells were cultured for another 72 hours. After 72 hours, cells were examined under a fluorescence microscope (CKX41; Olympus, Tokyo, Japan) to quantify GFP expression in the lentivirus particles. Cells were then trypsinised and subjected to flow cytometry analysis to determine lentiviral titer. The functional titer of lentivirus was calculated using the percentage of GFP-positive cells within the range of 1-30%. The titer was calculated according to the following formula:

TITER = (F × C × D)/0.025

F: frequency of GFP-positive cells (percentage divided by 100);

C: average number of cells per well on the day of transduction;

D: coefficient of lentivirus dilution (50X, 250X, 1250X, 6250X etc.);

V: 0.025 ml/25 ul (volume of inoculum).

### Lentivirus-mediated transduction in A549 cells

Lentivirus-mediated *RARβ* shRNA was transduced into A549 parental, putative CSCs (A549 CD166^+^EpCAM^+^) and putative non-CSCs (A549 CD166^-^EpCAM^-^) with a multiplicity of infection (MOI) of 5. A day before transduction, cells were seeded into 6-well plates at a density of 5×10^4^ cells/well. On the transduction day, the culture medium was replaced with a serum-free medium containing Polybrene (8 µg/ml). After 4-6 hours of transduction, a medium containing 10% FBS was added to the cells and continued cultured for another 72 hours. Then the cells were examined under a fluorescence microscope. Cells were analysed, and GFP-positive cells were sorted using FACSAria Fusion flow cytometry with the non-transduced cells as a negative control. All downstream experiments involving Lentivirus-mediated *RARβ* shRNA (Lv-sh*RARβ*) in A549 parental and putative CSCs and non-CSCs were conducted using isolated GFP+ cells.

### Real-time PCR

Total RNA from approximately 2×10^6^ cells was isolated using Simply P Total RNA Extraction Kit (Bioflux, China) according to the manufacturer's instructions. The extracted RNA's concentration and purity were determined using a Nanodrop ND1000 spectrophotometer (Agilent Technologies, Santa Clara, CA, USA). The cDNA was synthesised from total RNA (1 µg) using the SensiFast cDNA synthesis kit (Bioline BioCat GmbH, Heidelberg, Germany) according to the manufacturer's instructions. Quantitative real-time PCR (RT-PCR) reaction was prepared using QuantiNova SYBR Green PCR Kit (Qiagen, Hilden, Germany) for cancer-related genes (Table 1) and Taqman® Gene Expression Master Mix (Applied Biosystems, Thermo Fisher Scientific, Foster City, USA) for the expression of stem cells transcription factors (Table 2). The RT-PCR reaction was performed on ABI StepOnePlus™ PCR System (Applied Biosystems) with the following procedure: 95 °C for 4 min, 40 cycles of 95 °C for 15 sec, 60 °C for 30 sec, and 72 °C for 30 sec. The relative expression of the genes was calculated using the 2^-ΔΔCt^ analysis method. The expression of each gene was normalised to GAPDH as the housekeeping gene, and the relative expression of each gene was calculated using the 2^-ΔΔCt^ formula. The relative expression of each gene was relative to the expression of that gene in non-silencing cells.

### Migration Assay

The Lv-sh*RARβ* A549 parental, Lv-sh*RARβ* A549 CD166^+^EpCAM^+^ and Lv-sh*RARβ* A549 CD166^-^EpCAM^-^ cells and non-infected cells (control) were cultured at a density of 2×10^5^ cells/well in 24-well plate in RPMI 1640 complete medium overnight to reach 90% confluency. The next day, the cells were treated with 10 ng/ml colcemid for 2 hours for cell synchronisation. After incubation, a scratch was made using a sterile 200 µl pipette tip, and the scratch area was gently washed twice using PBS to remove detached cells. Cells were then incubated with 500 µl complete medium for 72 hours. Images of the migrated cells (eight fields of each well) were captured using relief contrast microscopy at ×100 magnification (OlympusIX 71; Olympus, Tokyo, Japan) at 24, 48, and 72 hours. The number of cells that migrated into the wound area was evaluated using the formula: Percentage of migrated cells = [initial scratch area (0 hr) - final scratch area (72 hr)]/initial (0 hr) × 100.

### Proliferation Assay

The Lv-sh*RARβ* A549 parental, Lv-sh*RARβ* A549 CD166^+^EpCAM^+^ and Lv-sh*RARβ* A549 CD166^-^EpCAM^-^ and non-infected cells (control) were cultured at a density of 5×10^3^ cells/well in 96-well plate in RPMI 1640 complete medium. The cells were cultured for 24, 48, and 72 hours, and the experiment was done in triplicates. Presto blue reagent (10 µl) (Invitrogen, Thermo Fisher Scientific, CA, USA) was added to each well after specific incubation time points and was incubated for 30 minutes in the dark at 37 °C. The samples were measured using a microplate reader at 590 nm.

### Cell Cycle Assay

The Lv-sh*RARβ* A549 parental, Lv-sh*RARβ* A549 CD166^+^EpCAM^+^ and Lv-sh*RARβ* A549 CD166^-^EpCAM^-^ and non-infected cells (control) were cultured in 6-well plate at a density of 3×10^5^ cells/well until reach 80 % confluency. The cells were then trypsinised and washed with ice-cold PBS. Cells were then fixed with pre-cold 75 % ethanol overnight at 4 °C. Samples were rewashed with ice-cold PBS twice and incubated with PBS containing RNAse (Bio Basic, New York, USA) and propidium iodide (PI) (Invitrogen) at 4 °C overnight in the dark. Cell cycle progression was analysed using a FACS Canto flow cytometer (BD Biosciences).

### Clonogenic assay

Briefly, Lv-sh*RARβ* A549 parental, Lv-sh*RARβ* A549 CD166^+^EpCAM^+,^ and Lv-sh*RARβ* A549 CD166^-^EpCAM^-^ and non-infected cells (control) were trypsinised, counted, and seeded for colony formation assay. One thousand cells were seeded in each well of a 6-well plate. After 7 days of incubation, the colonies were washed with PBS and fixed with 10% neutral buffered formalin for 10 min, followed by staining with 0.5% crystal violet solution (Sigma-Aldrich, St. Louis, MO, USA) for 30 min in the dark at room temperature. The numbers of colonies were manually counted, as previously described 7, 33.

### Sphere Forming Assay

The Lv-sh*RARβ* A549 parental, Lv-sh*RARβ* CD166^+^EpCAM^+^ and Lv-sh*RARβ* A549 CD166^-^EpCAM^-^ and non-infected cells (control). Briefly, the cells were re-suspended in a ratio of 1:1 (v/v) of growth factor-reduced Matrigel (BD Biosciences) attachment plate (Corning, Inc., Oneonta, NY, USA). The sphere was grown in serum-free DMEM-F12 (Gibco) supplemented with 20 ng/ml of epidermal growth factor (EGF), 10 ng/ml of basic fibroblast growth factor (bFGF), 1% B27 supplement (Invitrogen), and 1 % antibiotic-antimycotic (Gibco). The size and number of the formed spheroids were assessed after 21 days of culture using inverted phase-contrast microscopy (Olympus).

### Osteogenic and Adipogenic Differentiation Assay

Cells were differentiated into adipogenic and osteogenic lineages using Stempro® Adipogenesis and Osteogenesis differentiation kit (Gibco). Briefly, 6×10^4^ cells/well were cultured onto 24-well plate for 48-72 hours until they reached 100% confluency (for osteogenic differentiation) and 80% confluency (for adipogenic differentiation). The growth medium was then replaced with osteogenic or adipogenic induction medium, whereas for control (no induction), a complete RPMI growth medium was used instead. Cells were incubated for 21 days for osteogenic differentiation, and 14 days for adipogenic differentiation. The medium was replaced every three days. The capability of cells to differentiate into osteogenic was determined by deposition of calcium visualised by staining with 2% Alizarin Red S (Sigma-Aldrich), while the formation of adipocytes determined the capability of cells to differentiate into adipogenic visualised by staining with 0.3% Oil Red O (Sigma-Aldrich).

### Microarray analysis

#### RNA extraction, cDNA synthesis, purification, fragmentation, labelling and hybridisation

Total RNA was extracted from cells using the RNAeasy Kit (Qiagen). The extracted RNA's concentration and purity were determined using Nanodrop® ND1000 spectrophotometer, and the RNA integrity number (RIN) was determined using Bioanalyzer 2100 (Agilent Technologies). Only RNA with RIN >7 was used for amplification and hybridisation. cDNA library was generated from 1.5 µg of total RNA using the ApplauseTM WT-Amp ST System (Nugen Technologies, Inc., San Carlos, USA) following the manufacturer's protocol. The seven-step amplification process produced ST-cDNA, which was further purified using the MinElute Reaction Cleanup Kit (Qiagen). The yield and purity of the ST-cDNA were measured using the Nanodrop® ND1000 spectrophotometer. The A260:A280 ratio had to be more than 1.8, and the concentration must be in the range of 2 to 2.5 µg for the ST-cDNA to be hybridised to the array. The purified ST-cDNA was then fragmented and labelled with biotin (Nugen Technologies). Biotin-labelled fragmented ST-cDNA was hybridised to oligonucleotide probes on Affymetrix GeneChip® 1.0 ST arrays (Affymetrix, Santa Clara, USA) followed by washing and staining using the GeneChip® Hybridisation Wash and Stain Kit (Affymetrix). For each array, 2-2.5 µg of the fragmented biotin-ST-cDNA was hybridised to the array for 17 h at 45 °C in a rotating hybridisation oven. The array was stained on Affymetrix Fluidics Station FS450. The arrays were scanned with an Affymetrix Scanner 3000, and data were obtained using the GeneChip® Operating Software. The microarray experiment was performed using three biological replicates for each sample.

#### Data processing and analysis

Microarray data analysis was performed using Affymetrix Transcriptomic Analysis Console (TAC) software. The CEL files were subjected to background correction, summarisation and normalisation using Robust Multiarray Analysis (RMA). Statistical analysis using ANOVA was conducted by comparing the FC of cells with *RARβ* knockdown with normal cells (without silencing) to identify the significantly regulated genes in each group (Table 3). The probes/genes were then filtered based on p-value and FC. Probes/genes with *p* < 0.05 and FC > 2.0 were assumed to be significantly regulated.

#### Functional enrichment analysis

Functional enrichment analysis was performed using DAVID (http://david.abcc.ncifcrf.gov/) 34; 35. Significantly regulated genes (FC > 2; *p* < 0.05) from each group were submitted to DAVID. The list of differentially expressed genes (DEGs) was analysed for the enrichment in GO biological process and KEGG pathway.

## Results

### Lentivirus-mediated RNAi inhibits *RARβ* expression

**Figure 1:**
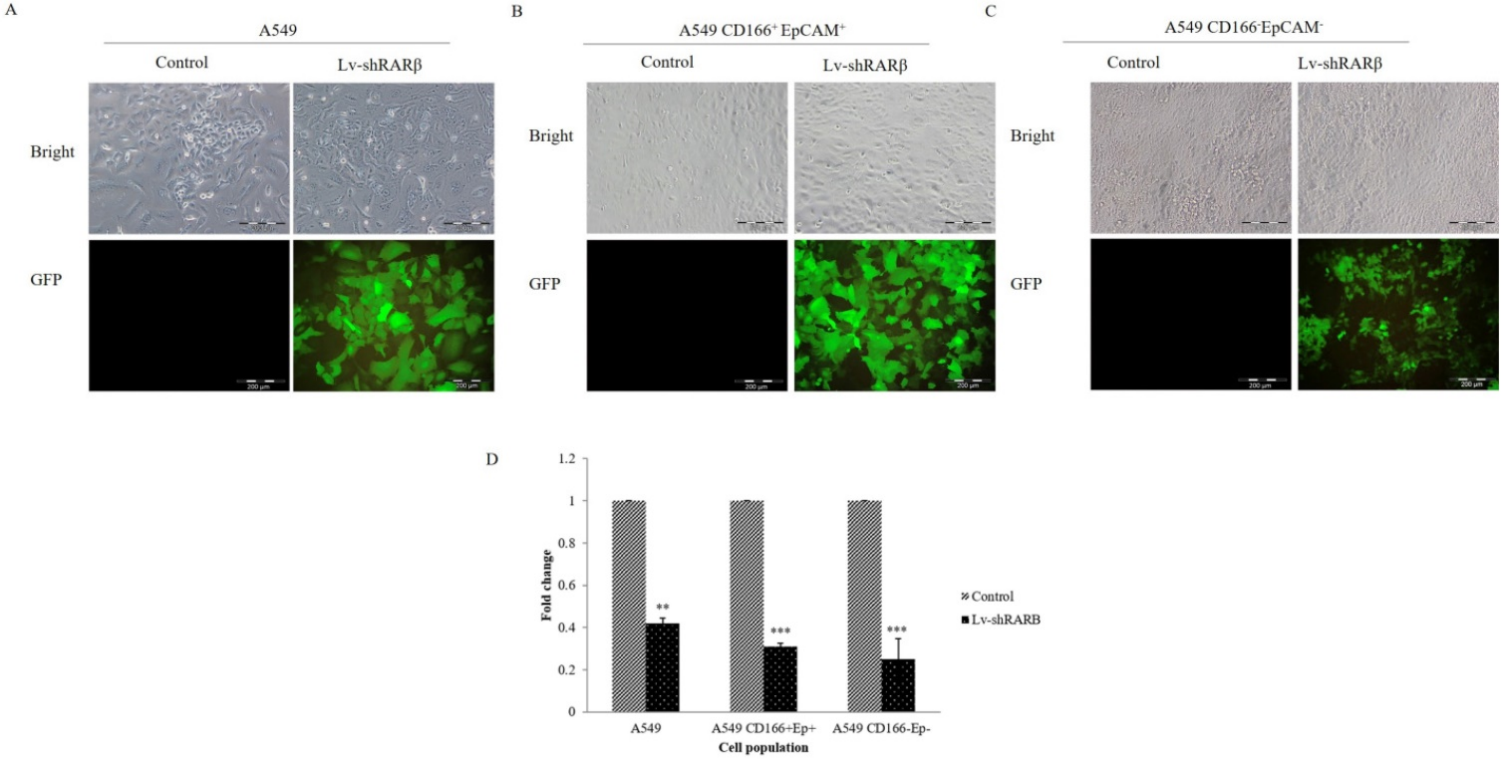
** RNAi of *RARβ* expression mediated by lentivirus.** Representative image of A549 parental (A) A549 CD166^+^EpCAM^+^ cell, (B) A549 CD166^-^EpCAM^-^, (C) transduced with lentiviral-mediated shRARβ. *RARβ* knockdown efficiency was measured by real time PCR (D). **p <0.01, ***p <0.001.

A549 parental, A549 CD166^+^EpCAM^+^ and A549 CD166^-^EpCAM^-^ were transduced with lentivirus containing *RARβ* shRNA and GFP reporter with an MOI of 5. More than 80% A549 and CD166^+^EpCAM^+^ cells and approximately 60% of A549 CD166^-^EpCAM^-^ cells were transduced by lentivirus as assessed by fluorescence expression of GFP reporter protein (Figure 1). The knockdown efficiency was determined by real-time PCR. Lentivirus-mediated RNAi caused a significant decreased of endogenous *RARβ* mRNA expression in A549 (60% decreased), CD166^+^EpCAM^+^ (70% decreased) and A549 CD166^-^EpCAM^-^ (75% decreased) (Figure 1).

### *RARβ* knockdown regulates other cancer-related gene expressions

**Figure 2:**
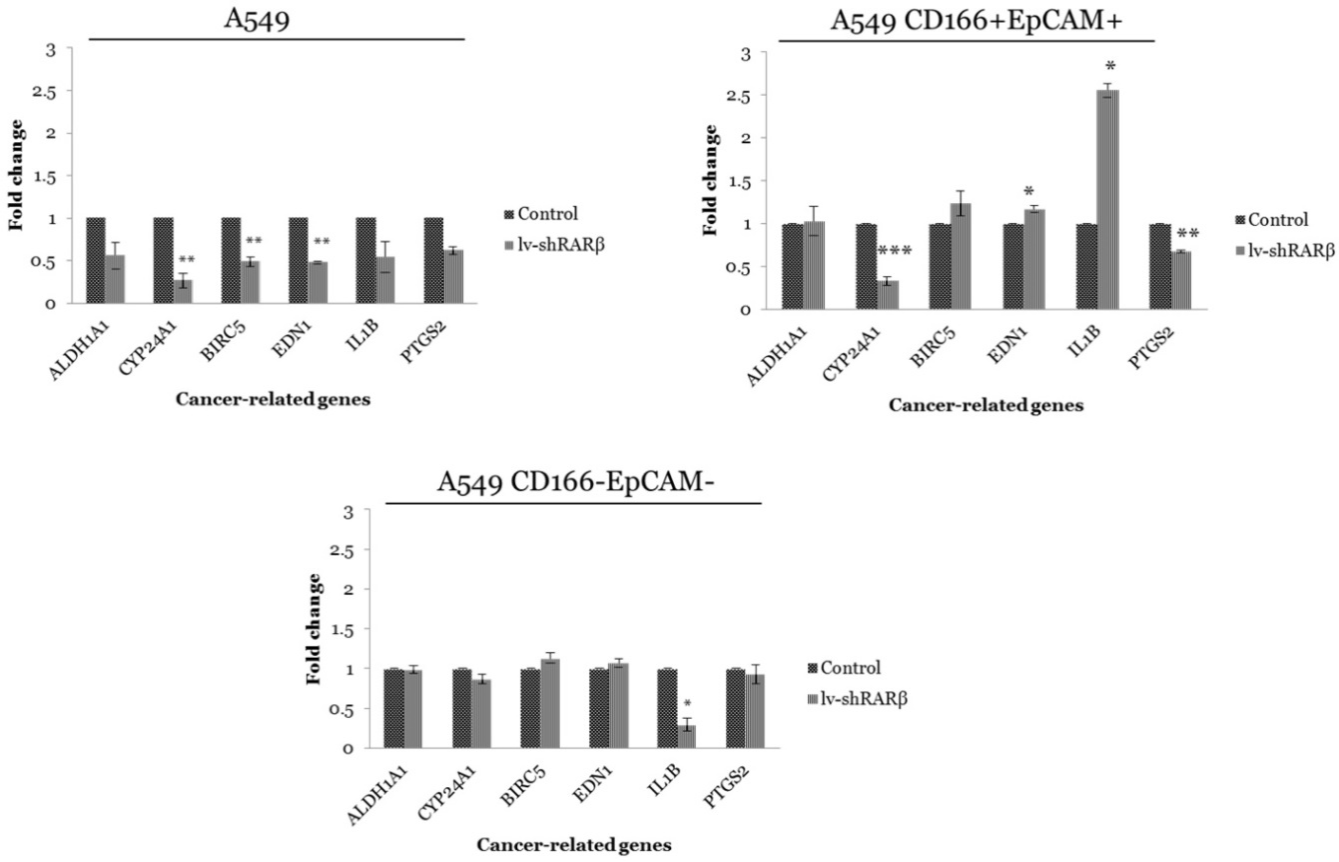
** Gene expression of cancer-related genes in control and lenti-shRARβ.** A) A549 cell, B) A549 CD166^+^EpCAM^+^ and, C) A549 CD166^-^EpCAM^-^. Down-regulation of all cancer-related genes was detected in A549-lentiviral-mediated RNAi (lenti-shRARβ) compared to the control group. The PCR reaction without mRNA template served as the negative control. The relative expression of the target genes was normalised to the housekeeping gene, *GAPDH*. The X-axis shows the target genes and the Y-axis shows the fold change. The error bars represent the standard deviation within the triplicates. *p<0.05, **p<0.01, ***p<0.001.

The effect of *RARβ* silencing was measured on the expression of six carcinogenesis-related genes, *ALDH1A1*, *CYP24A1*, *BIRC5*, *EDN1*, *IL-1β,* and *PTGS2,* and the result is shown in Figure 2. In parental A549 cells, all six genes were down-regulated where the significant reduction (*p*<0.01) were seen in the expression of *CYP24A1*, *BIRC5* and *EDN1* following *RARβ* silencing (Figure 2). In putative CSCs A549 CD166^+^EpCAM^+^, significant down-regulation of *CYP24A1* (*p*<0.001) and *PTGS2* (*p*<0.01) were detected. However, *EDN1* and IL-1β genes were significantly up-regulated (Figure 2). *RARβ* silencing does not affect the expression of all genes except the down-regulated expression of *IL-1β* (*p*<0.05) in putative non-CSCs A549 CD166^-^EpCAM^-^ (Figure 2).

### *RARβ* knockdown affects the expression of stem cell transcription factors

**Figure 3:**
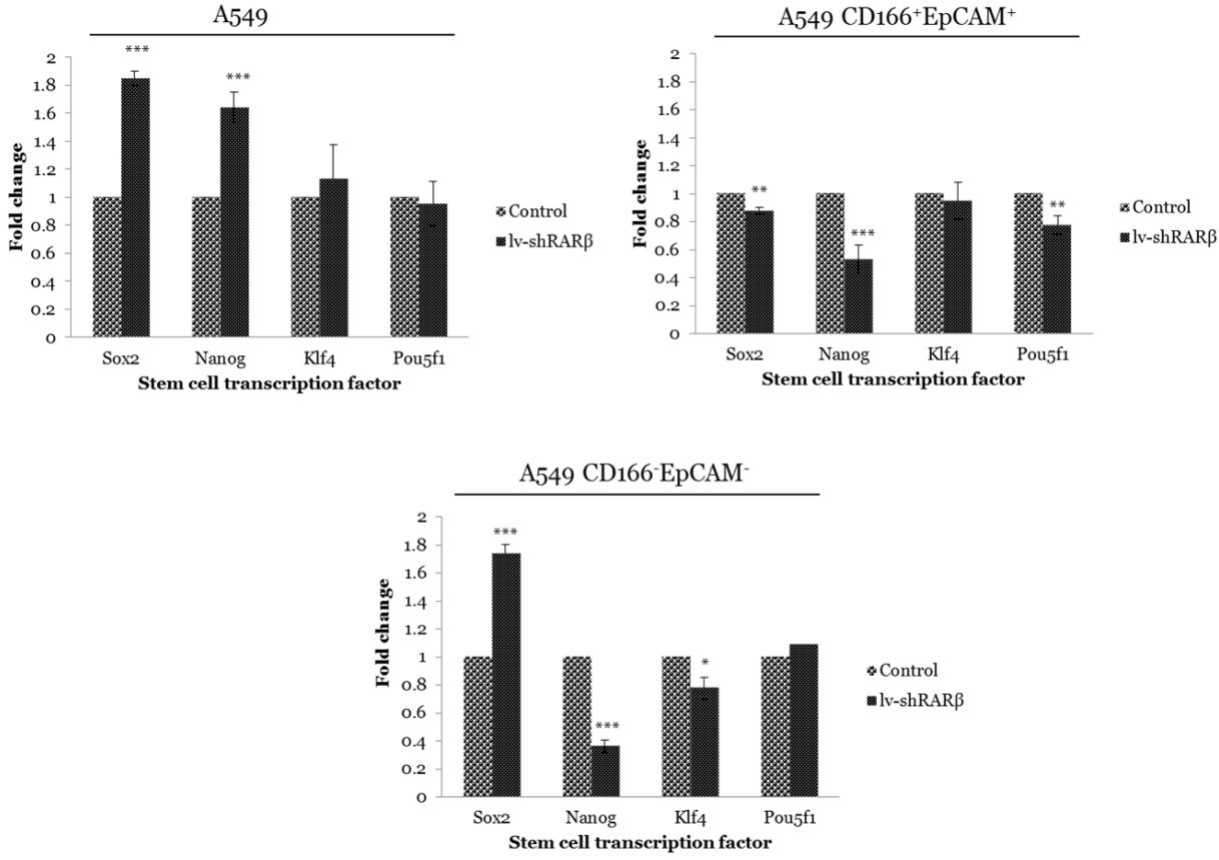
** Gene expression of stem cell transcription factor in control and the transduced groups (lenti-shRARβ).** A) A549 cell B) A549 CD166^+^EpCAM^+^ C) A549 CD166^-^EpCAM^-^. Detectable expression levels of the genes were found in all cell populations. The PCR reaction without template served as the negative control. The relative expression of target genes was normalised to the housekeeping gene, *GAPDH*. The X-axis shows the target genes and the Y-axis shows the fold change. The error bars represent the standard deviation within the triplicates. *p<0.05, **p<0.01, ***p<0.001.

The effect of *RARβ* silencing on CSCs was measured by analysing the expression of stem cell transcription factors *Sox2*, *Nanog*, *Klf4* and *Pou5f1* using qRT-PCR. These transcription factors are involved in maintaining normal stem cell pluripotency 36. Silencing of *RARβ* in A549 parental cells caused significant up-regulation of *Sox2* and *Nanog* (*p*<0.001) compared to the non-silencing A549 cells (Figure 3). Contrary, the expression of *Sox2*, *Nanog* and *Pou5f1* were significantly down-regulated (*p*<0.01 and *p*<0.001 respectively) in *RARβ* shRNA A549 CD166^+^EpCAM^+^ as compared to non-silencing cells (Figure 3). Similarly, significant down-regulation of *Nanog* (*p*<0.001) and *Klf4* (*p*<0.05) were detected in *RARβ* shRNA A549 CD166^-^EpCAM^-^ (Figure 3). These results suggest that the silencing of *RARβ* resulted in reducing the stemness characteristics in A549 parental but not in A549 putative CSCs and non-CSCs.

### Silencing of *RARβ* decreases self-renewal capacity in A549 parental

**Figure 4:**
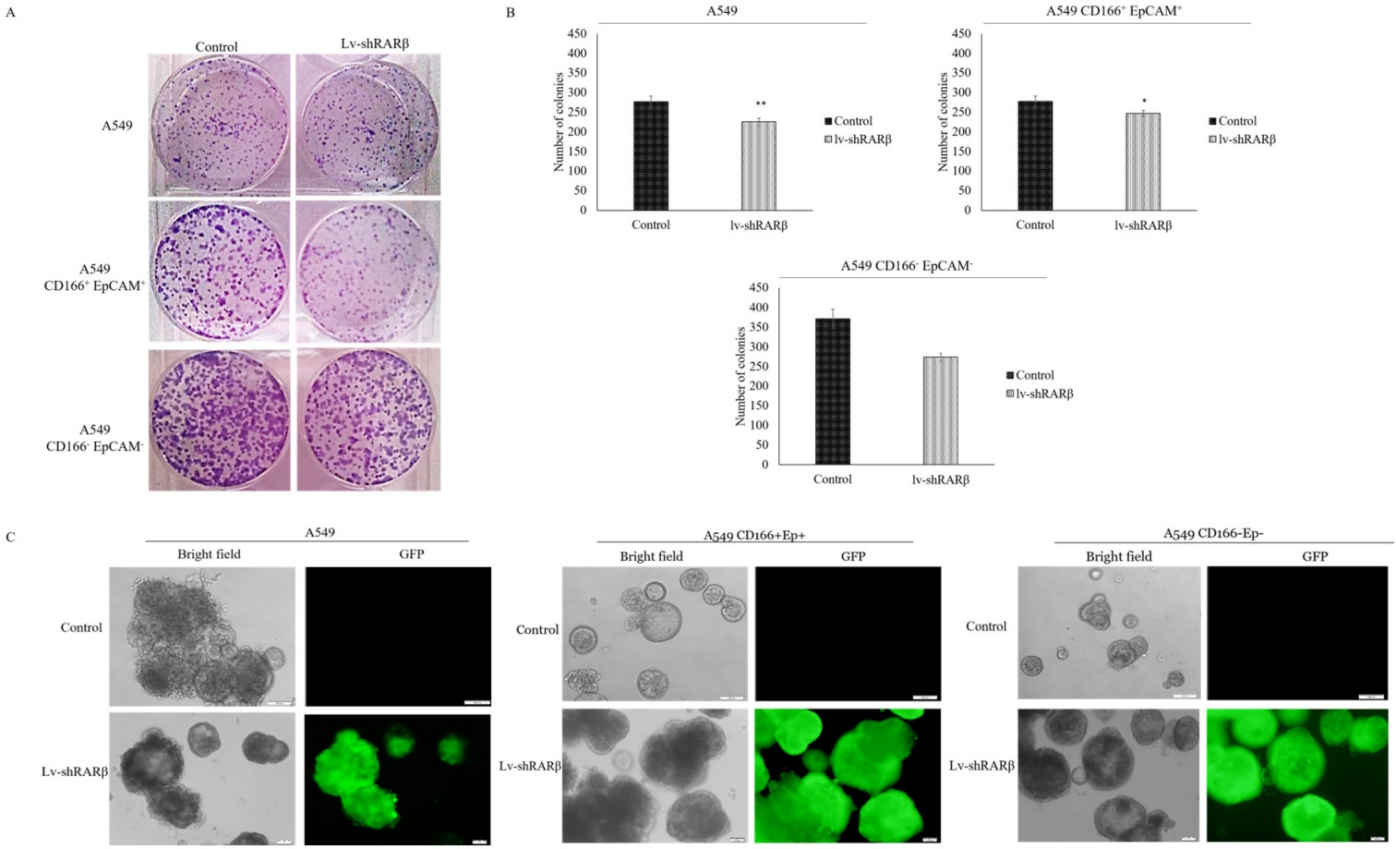

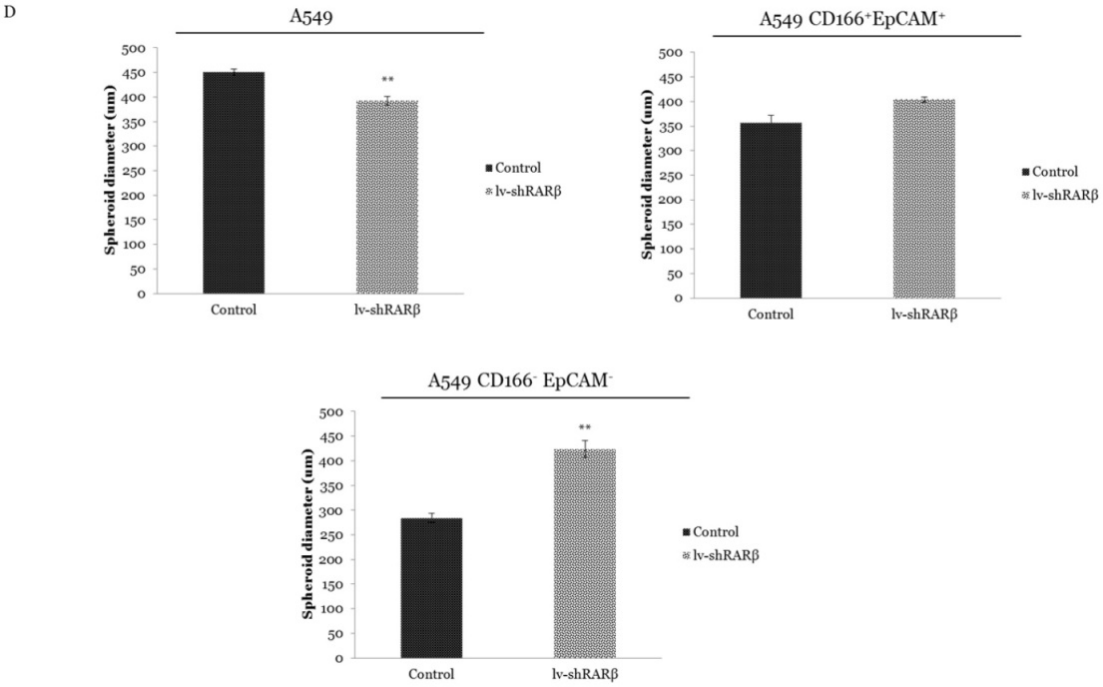
** Effect of *RARβ* knockdown on the self-renewal capacity of A549, A549 CD166^+^EpCAM^+^ and A549 CD166^-^EpCAM.** A) Representative images of colony forming assay after 7 days of incubation. B) The average number of colonies of different groups. C) Representative image of sphere forming assay after 21 days of culture. D) Average of spheroid diameter of different groups*p<0.05, **p<0.001.

The effect of *RARβ* silencing on CSCs was further measured on self-renewal characteristic, which was evaluated using a colony-forming assay for short term evaluation and sphere-forming assay for long term evaluation. The colony-forming efficiency of A549 parental, A549 CD166^+^EpCAM^+^ and A549 CD166^-^EpCAM^-^ were significantly reduced following *RARβ* silencing (*p*<0.05 and *p*<0.001 respectively) (Figure 4). This result was validated by sphere-forming assay. The result shows that the sphere size of *RARβ*-silenced A549 parental cells was significantly decreased (*p*<0.01) as compared to non-silencing A549 cells (Figure 4). However, in A549 CD166^+^EpCAM^+^ and A549 CD166^-^EpCAM^-^ cells population, *RARβ*-silenced cells formed larger spheres than non-silencing cells (Figure 4). This result suggests that knockdown of *RARβ* reduced the self-renewal capacity in A549 parental cell, putative CSCs, and non-CSCs for the short term period, but the self-renewal capacity of putative CSCs and non-CSCs were increased when cultured for a long time.

### Silencing of *RARβ* increases differentiation capability in A549 parental and A549 CSCs

**Figure 5:**
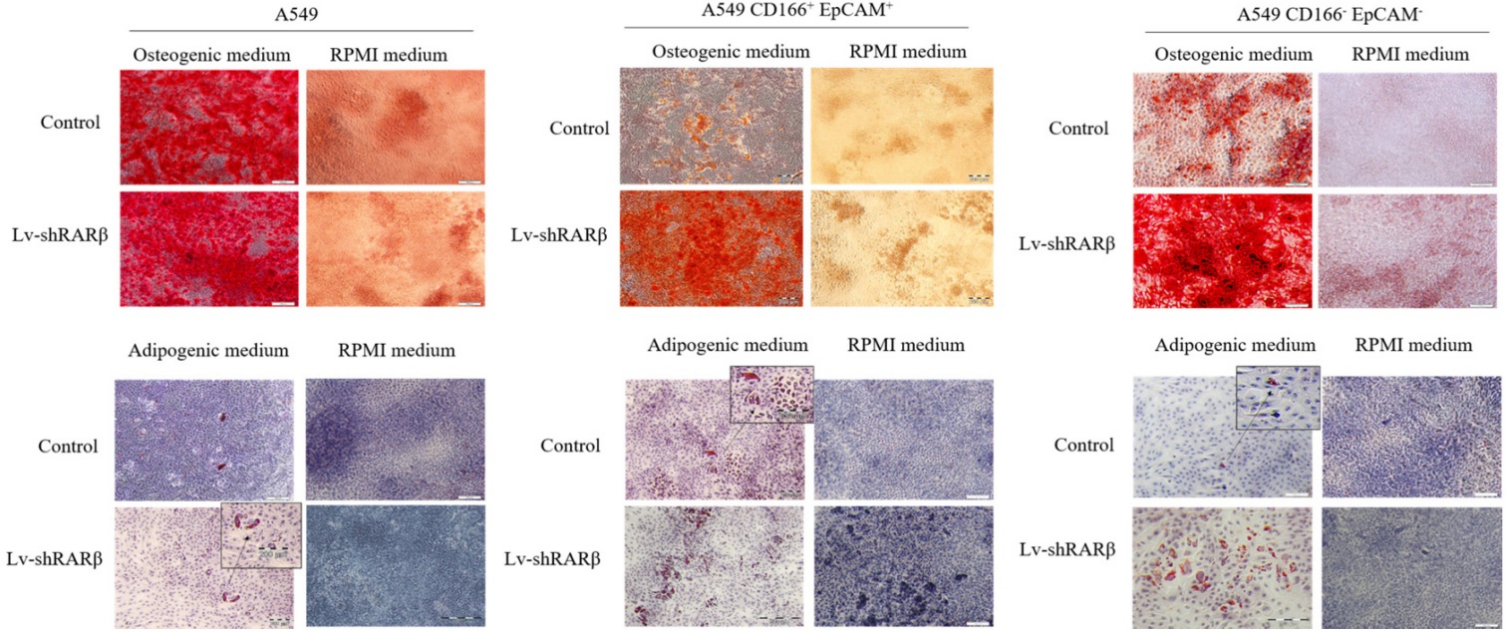
** Effect of *RARβ* knockdown on the differentiation capacity of A549 parental, A549 CD166^+^EpCAM^+^ and A549 CD166^-^EpCAM^-^.** (A) Osteocyte differentiation after 21 days of culture. Osteocytes were stained with alizarin Red S (B) Adipocyte differentiation after 14 days of culture, stained with Oil Red O staining.

Another characteristic of stem cells is the cells' capability to differentiate into three lineages; adipocytes, osteocytes, and chondrocytes. In this study, the cells' differentiation capacity was evaluated based on their ability to differentiate into adipocytes and osteocytes lineages. All three cell populations; A549 parental, A549 CD166^+^EpCAM^+^ and A549 CD166^-^EpCAM^-^ showed that *RARβ* silenced and non-silencing cells could form osteocytes and adipocytes (Figure 5). However, we found that more adipocytes and osteocytes were formed following the silencing of *RARβ* in all three cell populations; A549 parental, A549 CD166^+^EpCAM^+^ and A549 CD166^-^EpCAM^-^ suggesting that knockdown of *RARβ* triggered the differentiation capacity of A549 parental, A549 putative CSCs and A549 non-CSCs.

### Silencing of *RARβ* decreases A549 parental cells migration

**Figure 6:**
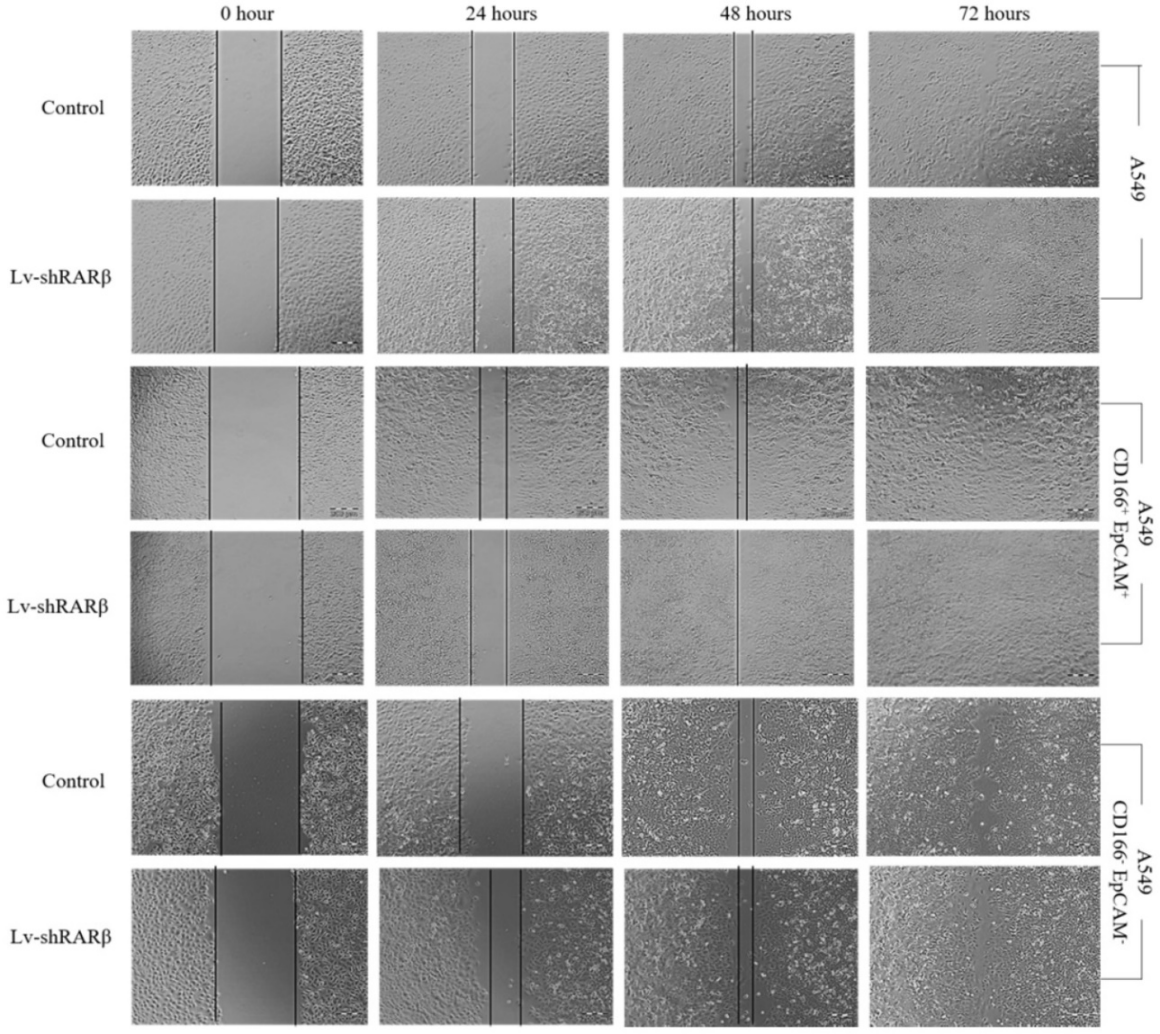
Effect of *RARβ* knockdown on the migration capacity of A549 parental, A549 CD166^+^EpCAM^+^ and A549 CD166^-^EpCAM^-^. The scratches were made on cells monolayer. The image of wound area were taken at 0, 24, 48 and 72 hours.

Stem cells also have the ability to migrate. The lentiviral-mediated RNAi (lenti-sh*RARβ*) cells migrated slower than the control, suggesting that the silencing of *RARβ* decreased the migration ability in A549 parental cells (Figure 6). However, in A549 putative CSCs and A549 non-CSCs, the lentiviral mediated RNAi (lenti-sh*RARβ*) cells had a higher mi rate than the control. Our results indicate that silencing *RARβ* decreases the migration rate in A549 parental cells, but not in putative CSCs and non-CSCs.

### *RARβ* depletion leads increase cells growth and G0/G1 cell cycle arrest in A549 and A549 CD166^+^EpCAM^+^

**Figure 7:**
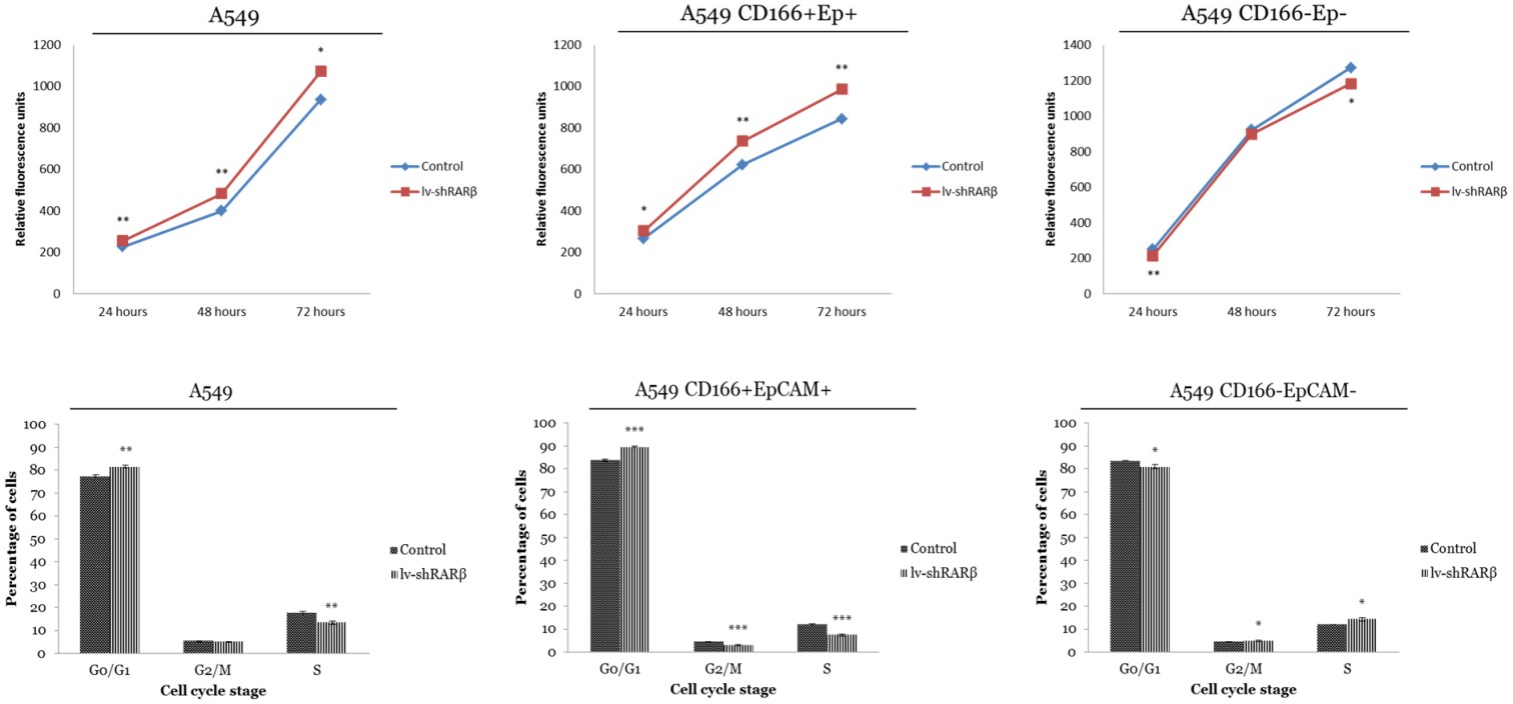
** Effect of *RARβ* knockdown on growth and cell cycle of A549 parental, A549 CD166^+^EpCAM^+^ and A549 CD166^-^EpCAM^-^.** The upper figure represent the growth of the cells as assess by presto Blue and the lower figure shows the Effect of *RARβ* knockdown on cell cycle progression of A549 parental, A549 CD166^+^EpCAM^+^ and A549 CD166^-^EpCAM^-^. Cells were blocked in the G0/G1 phase in the lenti-shRARβ. *p<0.05, **p<0.001, ***p<0.0001.

The effect of *RARβ* silencing on cell growth and cell cycle was analysed. The result indicates that the *RARβ* silencing has increased the growth rate of A549 parental and putative CSCs (Figure 7). For cell cycle analysis, in A549 parental cells, a higher percentage of cells accumulated in the G0/G1 phase of the cell cycle (81.61 ± 0.65%) after lenti-sh*RARβ* transduction, compared to the control (77.26 ± 0.69%) (Figure 7). The percentage of cells in the S phase was markedly decreased after lentivirus transduction (13.38 ± 0.18%) compared to control (17.44 ± 0.11%). The same pattern was seen in A549 CD166^+^EpCAM^+^, where the percentage of cells arrested at G0/G1 was higher in the transduced group (89.69 ± 0.26%) compared to the control group (83.81 ± 0.39%) (Figure 7). However, in A549 CD166^-^EpCAM^-^ cells, there was an accumulation at the S phase of the cell cycle (14.33 ± 0.78%) after lenti-sh*RARβ* transduction, compared with the percentage in the control group (11.93 ± 0.03%) (Figure 7). These results suggest that knockdown of *RARβ* might suppress the growth of A549 and A549 CD166^+^EpCAM^+^ via cell cycle arrest.

### The effect of *RARβ* silencing on global gene expression profiles

**Figure 8:**
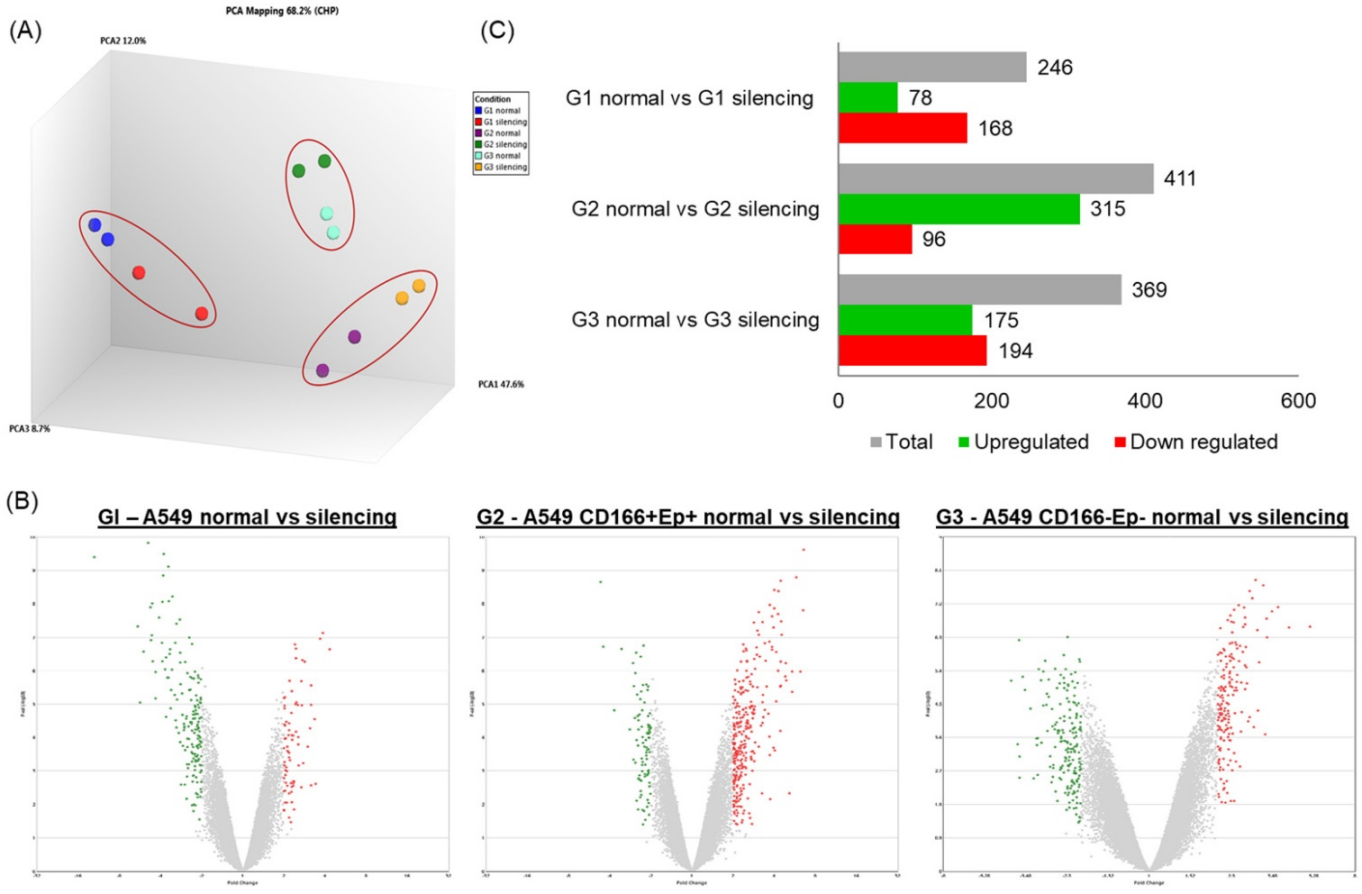
** Analysis of differentially regulated genes.** (A) A principle component analysis (PCA) was the initial analysis performed for all samples using the Transcriptome Analysis Console 3.0 (Affymetrix®). (B and C) Genes having the cut-off criteria of FC ≥+2 or ≤-2, and a p-value ≤0.05 are considered significantly regulated. The bar chart and volcano plot show the number of significantly up or down regulated genes.

Microarray analysis was performed to elucidate the global gene expression changes following *RARβ* gene silencing in A549 parental, putative CSCs and non-CSCs cells. Principal component analysis (PCA) was performed for all identified genes from the microarray to identify the samples' variation. The PCA analysis showed significant separation between different cell subtypes (PCA1: 47.6% variance) (Figure 8). There was only a 12% variance (PCA) between cells with *RARβ* silencing compared to the control. This result indicates a clear distinction between cells with *RARβ* silencing and the non-silencing group (Figure 8).

The mRNA expression in cells with *RARβ* gene silencing was compared to mRNA expression in non-silencing cells (Table 3) to understand the changes in the global gene expression pattern following the silencing of *RARβ*. The differentially regulated genes (DEGs) were filtered by the volcano plot at *p*≤0.05 and fold change (FC) of ≥ +2.0 or ≤ -2.0 (Figure 8). In A549 parental cell (group 1), out of 246 genes, 78 genes (31.7%) were significantly up-regulated, and 168 genes (68.3%) were significantly down-regulated (Table 3; Figure 8). In putative CSCs group (A549 CD166^+^EpCAM^+^), out of 411 genes, 315 genes (76.6%) were up-regulated and 96 genes (23.4%) were down-regulated (Table 3; Figure 8). In putative non-CSCs group (A549 CD166^-^EpCAM^-^), out of 369 genes, 175 genes (47.4%) were up-regulated and 194 (52.6%) genes were down-regulated (Table 3; Figure 8).

**Figure 9:**
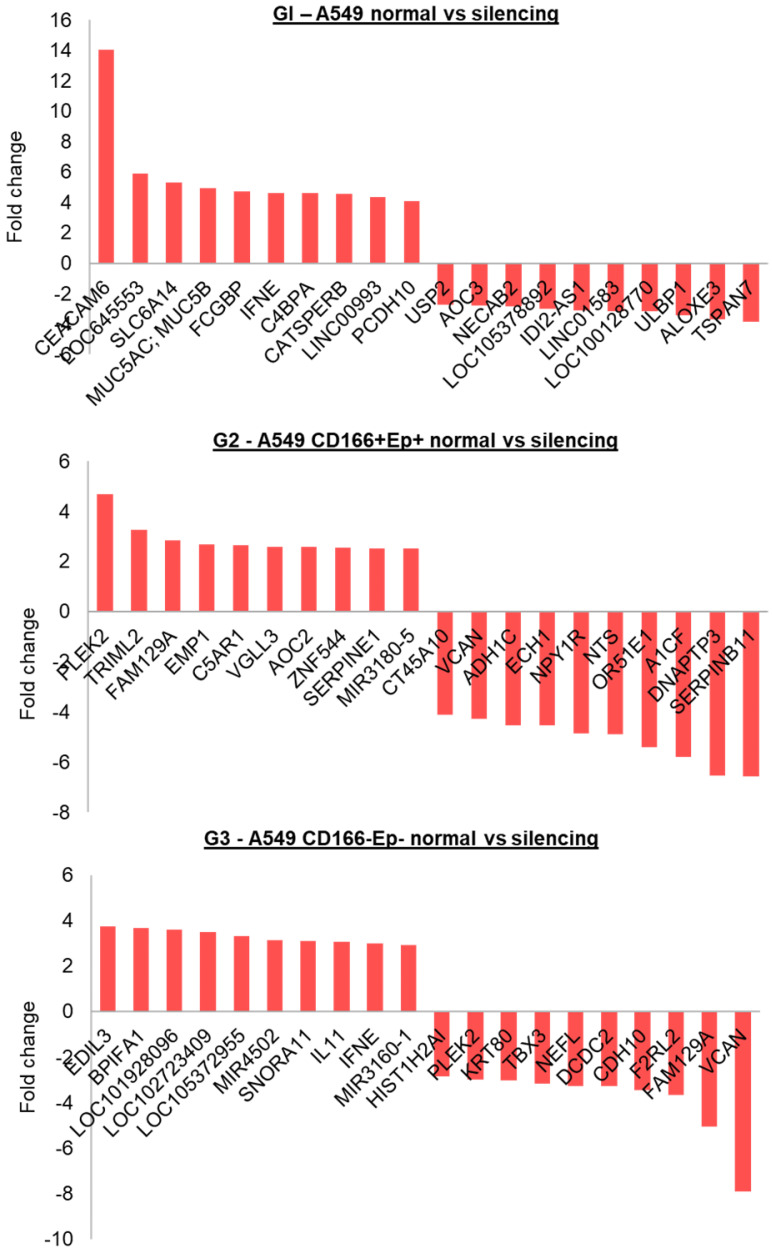
Top 10 up and down regulated genes within each group.

The top 10 genes that were significantly up or down-regulated are shown in Figure 9. In A549 parental (G1), the highest up-regulated gene was *CEACM6* (Fold change ~14), and the most down-regulated gene was *TSPAN7*. In A549 CSCs (G2), the highest up-regulated gene was *PLEK2* (Fold change ~5), and the most down-regulated gene was *DNAPTP3*. *EDIL3* and VCAN were the highest up-regulated and down-regulated genes in putative non-CSCs, respectively.

### GO biological process and KEGG pathways enrichment analysis of significantly expressed genes

The DEGs of each cell type were subjected to Gene Ontology (GO) and Kyoto Encyclopedia of Genes and Genomes (KEGG) pathways analysis using the Database for Annotation, Visualization, and Integrated Discovery (DAVID) program to better understand the biological processes and pathways. The analysis was separated between up-regulated and down-regulated genes.

**Figure 10:**
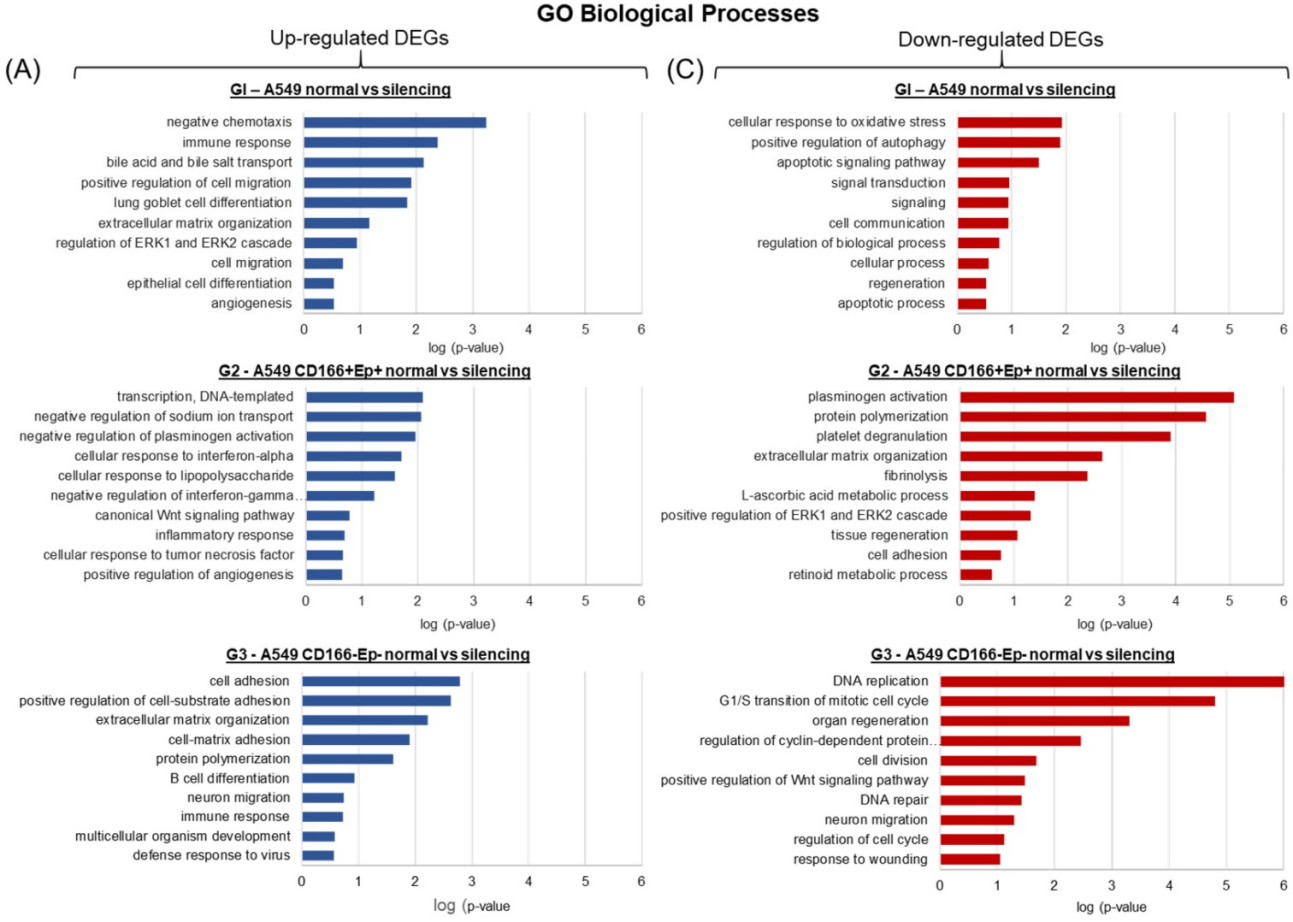
** Analysis of the biological processes affected by the DEGs.** Gene Ontology (GO) enrichment analysis of DEGs was performed using DAVID online bioinformatics tool 33; 34. The up- and down-regulated DEGs for each group were analysed separately and the ten most significantly enriched GO biological processes were selected to represent each group.

In A549 parental cells, the up-regulated DEGs were enriched in biological processes related to immune response, cell migration, cell differentiation and angiogenesis (Figure 10). In contrast, the down-regulated genes were involved in biological processes related to oxidative stress, apoptosis, signalling, cell communication and regeneration (Figure 10). The DEGs in A549 putative CSCs are involved in cytokine signalling processes such as interferon-α and interferon-γ and Wnt signalling pathways (Figure 10). In contrast, the down-regulated genes are involved in biological processes such as ERK signalling, tissue regeneration and retinoic metabolic process (Figure 10). The up-regulated genes in A549 putative non-CSCs are involved in cell adhesion, cell differentiation, neuron migration and immune response (Figure 10), while the down-regulated genes are involved in biological processes such as DNA replication and repair, cell cycle, cell division and Wnt signalling pathway (Figure 10).

**Figure 11:**
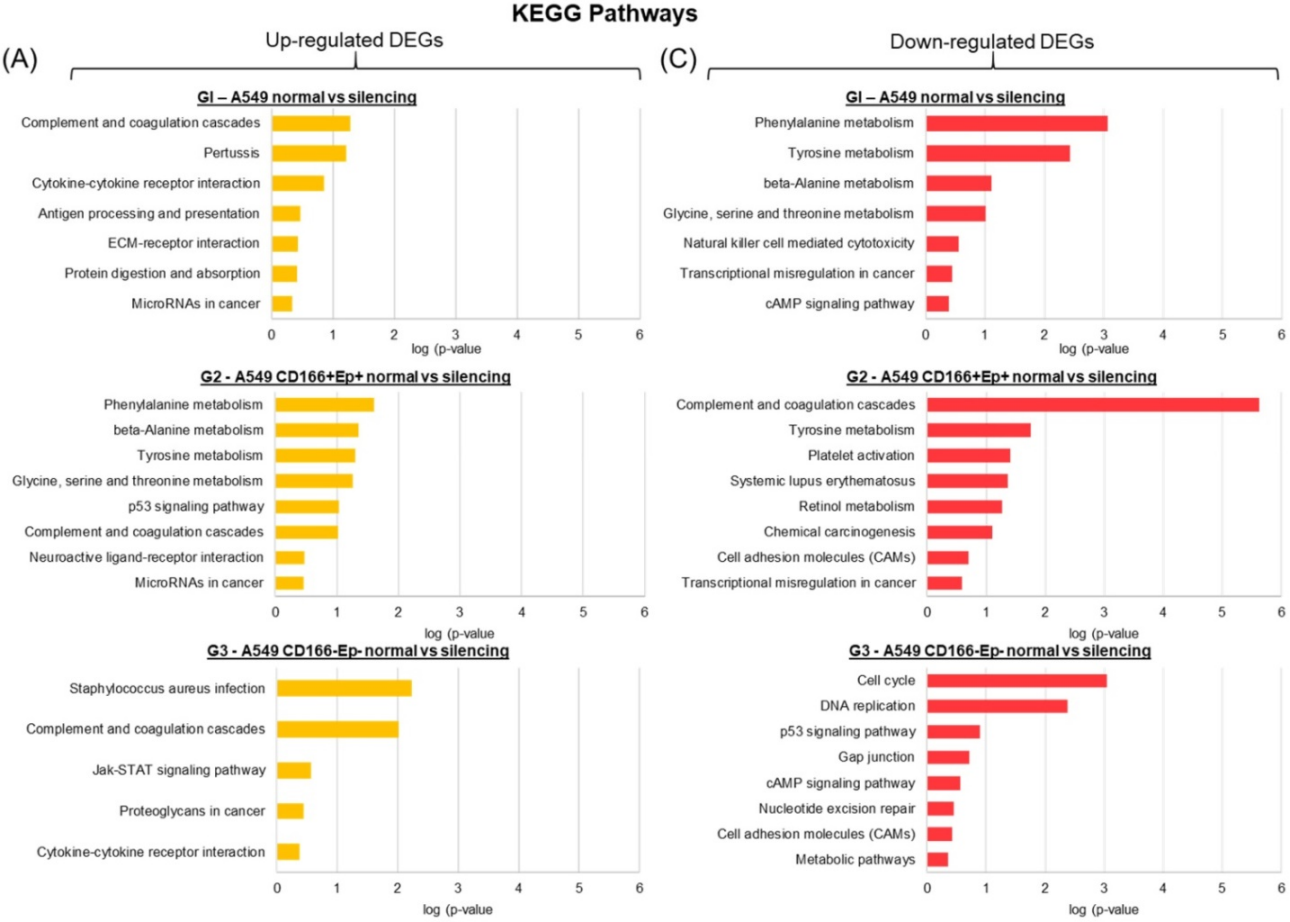
** KEGG pathway of all significantly up-regulated and down-regulated genes in A549 parental, putative CSCs and putative non-CSCs.** (A) Bar chart showing the enriched KEGG pathways for up-regulated genes and (B) Bar chart showing the enriched KEGG pathways for down-regulated genes.

For KEGG pathways, the up-regulated and down-regulated genes in all three cell types shared a few common pathways. For example, the complement and coagulation cascade pathway was enriched in up-regulated genes of all three cell types (Figure 11); cytokine-cytokine receptor interaction was enriched in A549 parental and non-CSCs (Figure 11), and the pathway of MicroRNAs in cancer was enriched in A549 parental and putative CSCs (Figure 11). The down-regulated genes in A549 parental cells and putative non-CSCs were enriched in the cAMP signalling pathway (Figure 11), while the down-regulated genes in putative CSCs and non-CSCs were enriched in cell adhesion molecules (CAMs) pathways (Figure 11). p53 pathway was significantly up-regulated in putative CSCs. However, in putative non-CSCs, this pathway was down-regulated (Figure 11).

### Main pathways involved in the specific DEGs of each cell type

**Figure 12:**
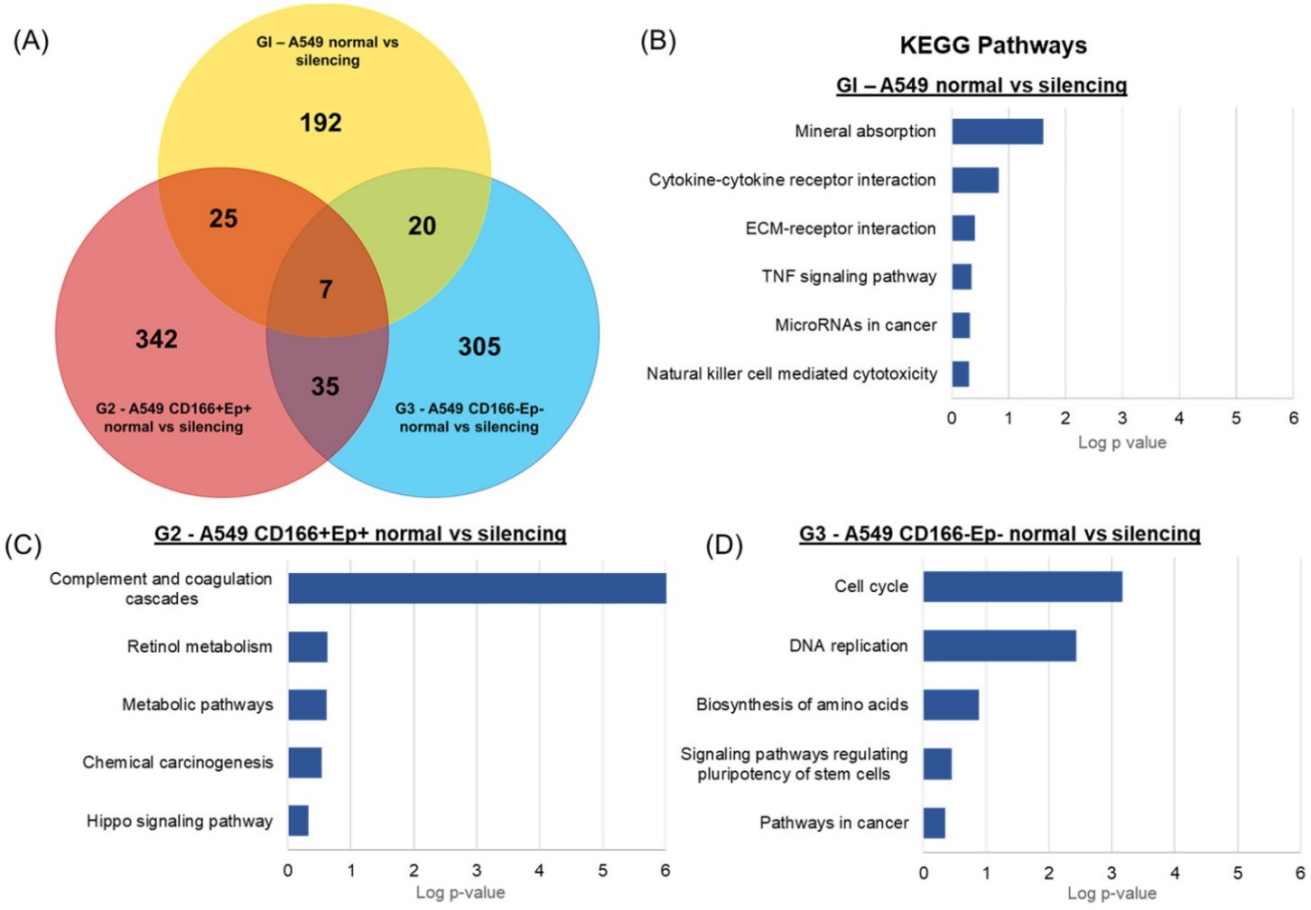
** KEGG pathway of specific DEGs in each cell phenotype.** (A) Venn diagram showing the number of DEGs overlapping between different cells types. Enriched KEGG pathways of (B) 192 DEGs specific to A549 parental cells, (C) 342 DEGs specific to A549 putative CSCs and (D) 305 DEGs specific to A549 putative non-CSCs analysed in DAVID bioinformatics tool.

Venn diagram analysis was performed to evaluate the common DEGs shared by A549 parental, putative CSCs and putative non-CSCs following *RARβ* gene silencing to compare the global expression changes (Figure 12). The analyses revealed only 7 DEGs were shared by the three groups (Table 4). 25 DEGs were common in A549 parental and putative CSCs, 20 DEGs were common in A549 parental and putative non-CSCs, and 35 DEGs were common in putative CSCs and non-CSCs group. Moreover, 192 DEGs were specific to A549 parental cells only, 342 to putative CSCs and 305 to putative non-CSCs.

For the KEGG pathway analysis, DEGs that were specifically regulated in A549 parental cells were involved in cancer-related KEGG pathways such as TNF signalling pathways (*CXCL1* and *CREB5*), ECM-receptor interaction (*ITGB4* and *COL5A2*), Natural killer cell-mediated cytotoxicity (*TNFRSF10D* and *ULBP1*) and MicroRNAs in cancer (*HMOX1*, *PLAU* and *MIR23B*) (Figure 12). DEGs in A549 CSCs that were enriched in KEGG pathways included complement and coagulation cascades pathway (*C5AR1*, *F5*, *FGA*, *FGB*, *SERPINE1*, *CFH* and *CFD*), retinol metabolism (*ADH1C* and *ADH6*), chemical carcinogenesis (A*DH1C* and *ADH6*) and Hippo signalling pathway (*SERPINE1* and *CDH*1) (Figure 12). Specific DEGs in non-CSCs cells showed enrichment in cancer-related pathways such as the cAMP signalling pathway (*ADCY3*, *ORAI1*, *HTR1D* and *GLI1*), basal cell carcinoma (*WNT11* and *GLI1*), p53 signalling pathway (*DDB2* and *SFN*) and pathways in cancer (*ADCY3*, *GNA11*, *WNT11*, *BIRC3*, and *GLI1*) (Figure 12). Moreover, stem cell-related pathways and signalling pathways regulating pluripotency of stem cells (*INHBB*, *TBX3* and *WNT11*) were also enriched in DEGs of A549 non-CSCs.

## Discussion

*RARβ* is a nuclear receptor protein that functions as a retinoic acid receptor. By binding to *RARβ*, retinoid can regulate cell growth, cell differentiation and cell apoptosis 1.* RARβ* gene has been reported to be silenced in most non-small cell lung cancer (NSCLC) and is associated with an increased risk of lung cancer 2. However, a recent study showed that this gene has dual lung cancer functions, as cancer suppressor and cancer promoter 3. Our previous study on transcriptomic profiling of lung CSCs using microarray analysis showed that the expression of *RARβ* was 2.4-fold higher in A549 than normal lung cells and 4.2-fold higher in putative CSCs compared to normal lung stem cells. Moreover, the expression of *RARβ* was 2-fold higher in A549 putative CSCs than A549 parental cells suggesting that high expression of *RARβ* might play an essential role in CSCs maintenance 7. To understand the role of *RARβ* in lung CSCs, cells were transduced with lentiviral-shRNA against *RARβ* to establish the knockdown of the *RARβ* gene, and the effect of gene knockdown was assessed.

Following *RARβ* silencing, the down-regulation of cancer-related genes, *ALDH1A1*, *CYP24A1*, *BIRC5*, *EDN1*, *IL-1β* and *PTGS2* in A549 parental cell suggest the importance of *RARβ* in controlling the expression of genes related to carcinogenicity. Down-regulation of *ALDH1A1*, *CYP24A1*, *BIRC5* and* EDN1* following *RARβ* silencing were consistent with the study done by Pappas et al., 25. However, the result was contradicted in the putative CSCs and non-CSCs populations, in which the *BIRC5* and *EDN1* were found to be highly expressed. *BIRC5* was known to play an essential role as an anti-apoptosis 4. Previous studies showed that *BIRC5* or known as survivin, was highly expressed in various cancer cells, including lung cancer (86% expression), prostate cancer (71% expression), ovary cancer (29-85%), breast cancer (71-90%) and gastric cancer (35-68%) whereas in normal adult tissues this gene was expressed at a low level 37. This result suggested that silencing of *RARβ* in A549 parental cell suppresses cancer cell growth by down-regulates the expression of *BIRC5* to the average level. On the other hand, the silencing of *RARβ* enhanced the expression of *BIRC5* in A549 CD166^+^EpCAM^+^ and A549 CD166^-^EpCAM^-^, suggesting that depletion of *RARβ* promotes the cancer cell growth via activation of the anti-apoptosis gene. The enhancement of the anti-apoptosis gene may lead to the escaping of cells from the body repairing system.

*EDN1* functions as a neo-angiogenesis and a mitogenic factor 6,11. A previous study showed that the silencing of *RARβ* leads to the downregulation of the *EDN1* gene (cite). However, our study showed the opposite result where silencing of *RARβ* resulted in overexpression of this gene. The up-regulation of *EDN1* in A549 CD166^+^EpCAM^+^ and A549 CD166^-^EpCAM^-^ was consistent with our findings in the migration assay where suppression of *RARβ* increased the migration ability of these cells. The enhancement of migration ability might occur via the up-regulation of *EDN1* expression 12. Our finding was consistent with a previous study on prostate cancer, which revealed that *EDN1* might modulate bone metastases from prostate cancer. Other studies also reported the effect of *EDN1* towards cell metastasis in hepatocellular, gastric and prostate cancer 38,39,40. This suggests that the silencing of *RARβ* enhances the cancer growth in A549 CD166^+^EpCAM^+^ and A549 CD166^-^EpCAM^-^ cell populations by *EDN1* overexpression of the *EDN1* gene which play important role in cancer metastasis.

In the A549 CD166^+^EpCAM^+^ population, the *IL-1β* gene, which responsible for the production of the pro-inflammatory cytokine, was highly up-regulated (2.5 fold higher) in *RARβ* silencing putative CSCs as compared to non-silencing cells. Over-expression of these genes are associated with cancer pathogenesis 16. For instance, overexpression* of IL-1β* in human malignant gliomas was associated with cancer cell proliferation, migration and invasion 40,41. The overexpression of *IL-1β* was consistent with the enhancement of proliferation and differentiation capability of the cells following the suppression of *RARβ*, indicating that the *IL-1β* expression regulates cellular activities such as cell proliferation and differentiation 19.

The effect of *RARβ* depletion on the expression of stem cell transcription factors - *Sox2, Nanog, Klf4* and *Pou5f1* was then evaluated. These transcription factors play an important role in maintaining stem cell pluripotency and are required for an efficient self-renewal capability of stem cells 20. In A549 parental cells, expression of *Sox2*, *Nanog* and *Klf4* were elevated in *RARβ*-silenced cells compared to the non-silenced cells. However, the expression of all four genes was down-regulated in A549 CD166^+^EpCAM^+^. Similarly, the expression of *Nanog* and* Klf4* were also down-regulated in *RARβ*-silenced A549 CD166^-^EpCAM^-^. The down-regulation of the stem cell transcription factors would reduce the self-renewal characteristics, but our result shows the opposite. Long-term self-renewal characteristics of *RARβ*-silenced putative CSCs were increased as compared to non-silenced cells as evidence by their ability to form bigger spheres size. The heterogeneity of cancer cells in parental cells containing a mixture of CSC populations has shown a clear indication that the silencing of RARβ might have affected other CSCs populations, but not the CSC that shown positive for CD166^+^EpCAM^+^ and CD166^-^EpCAM^-^. However, the contradict roles of *RARβ* in controlling the self-renewal capability of these populations of cells remain unclear.

Cell cycle assay also demonstrated that lentivirus-mediated *RARβ* knockdown inhibited the growth of A549 parental cells along with cell cycle arrest at the G_0_/G_1_ phase. However, growth inhibition could not be seen in A549 CD166^+^EpCAM^+^ and A549 CD166^-^EpCAM^-^. Based on results obtained, suppression of *RARβ* inhibits the growth of A549 parental cells. These results indicate that A549 parental cells are comprised of a heterogeneous cell population. It also suggests that inhibition of the stemness capacity of A549 cells occurs via targeting other cancer stem cell markers, instead of A549 CD166^+^EpCAM^+^ and A549 CD166^-^EpCAM^-^. On the other hand, the silencing of *RARβ* enhances the growth of A549 CD166^+^EpCAM^+^ and A549 CD166^-^EpCAM^-^. The contradictory roles of *RARβ* in sorted CSCs compared to parental cells require further investigation to identify other tumorigenic pathways that may regulate CSCs to maintain their stemness and capability despite the loss of *RARβ*.

Transcriptomic and pathway analyses using microarray was conducted to explore and understand the changes following *RARβ* gene silencing. Based on the data presented so far, data at the cellular level showed that silencing *RARβ* had a contradicting effect on parental A549 cells and CSCs. *RARβ* gene silencing reduced the tumorigenic characteristics of A549 parental cancer cells but increased the stemness characteristics of CSCs. Therefore, it is vital to understand the genes and pathways regulated in CSCs following *RARβ* silencing.

The microarray data showed that genes involved in cancer pathogenesis and cancer pathways were up-regulated in CSCs. Previous findings also showed that the suppression of the *RARβ* gene through hypermethylation of the *RARβ* gene promoter contributed to NSCLC pathogenesis 21. Moreover, several studies also reported that the suppression of *RARβ* in NSCLCs might be associated with lung carcinogenesis 22. The *RARβ* gene expression can cause RA-dependent and RA-independent apoptosis and growth arrest, mediated through RARα. *RARβ* protein results in the expression of a number of its target genes that mediate cell differentiation and death. Inactivation of *RARβ* contributes significantly to tumorigenesis of a variety of cancers including NSCLC 23. Restoration of *RARβ* function resulted in decreased tumorigenicity. For example, in lung cancer cells A549 and H460 treated with curcumin, the *RARβ* expression was increased at mRNA and protein level and decreased tumorigenicity 43.

Several genes were highly up-regulated in A549 CSCs following *RARβ* silencing, including *PLEK2, TRIML2, FAM129A, EMP1, C5AR1, VGLL3, AOC2, ZNF544, MIR3180-5* and* SERPINE1*. Among these genes, *TRIML2, FAM129A, EMP1,* and* SERPINE1* were involved in inducing tumorigenicity*.* FAM129A encodes for Niban, an anti-apoptotic protein that is overexpressed in many cancer 44. This protein prevents apoptosis by releasing Mdm2 protein, which stimulates the proteasomal degradation of p53 protein 45.

Moreover, *RARβ* silencing also induced an up-regulation of *EMP1* in putative CSCs. This gene was reported to be down-regulated in the cancer region compared to the adjacent normal epithelium at the primary tumour site 46. *EMP1* has been proposed as a marker for resistance in cancer therapies and related to poor prognosis. On top of that, the up-regulation of *EMP1* was shown to increase cancer cell migration and lead to metastasis 47. In order to understand the mechanisms of these genes to enhance CSCs stemness and promote tumorigenicity, further down-stream analysis needs to be performed. For instance, small molecule or chemical that functions to block these genes' function can be given to *RARβ* silenced CSCs. On top of that, the effect of *RARβ* silencing on other phenotype of CSCs could be further explored. The CSCs could be isolated using different approaches such as using different marker combination, selection based on chemo-resistant characteristics or enrichment of CSCs population by using 3D culture method.

The results revealed the cell specificity role of *RARβ,* where this gene act asa tumour suppressor gene in A549 parental cells and play the opposite role in A549 CSCs where in CSCs, this gene play role as a tumour promoter. Even though this study results show reliable findings, however, the findings of this study have to be seen in the light of some limitations. The laboratory findings of the present study could not reflect the *in vivo* status. Therefore, it would be highly recommended for animal study to be conducted to address this issue.

In the present study, PHBEC (Primary Human Bronchial/Tracheal epithelial cells) was used as control at the beginning of the present study to isolate cancer stem cells. However, this control cell line was not tested in *in vitro* analysis. It was due to our aim, to compare the effect of *RARβ* silencing between the parental cell lines with putative CSCs. Hence, the normal cell line was not tested for *in vitro* experiments. However, the present study has a limitation in terms of the lack of other cancer cell lines in this study. We have tried using another cell line, which was H2170 to represent squamous cell carcinoma. However, due to the difficulties in maintaining the cell, the experiment could not be performed using these cell lines. Thus, it would be suggested for the future to validate the present study's findings by using other NSCLC cell lines.

## Conclusion

In conclusion, the role of *RARβ* is cell-specific, where this gene acts as a tumour suppressor in A549 parental cells, yet enhances the stemness characteristic of CSCs. *RARβ* played a role as a regulatory gene as the silencing of this gene led to the downregulation of cancer-related genes in A549 parental cells. However, overexpression of cancer-related genes; *EDN1* and *BIRC5* in CSCs have been detected, suggesting that there is another networking pathway which triggering the activation of these genes which led to the enhancement of stemness characteristics of putative CSCs populations. The genes identified from microarray (*TRIML2, FAM129A, EMP1,* and* SERPINE1)* might be responsible for enhancing CSCs stemness following *RARβ* silencing. Understanding the roles of these genes, especially in regulating the stemness of CSCs in NSCLC, could lead to a future targeted therapy for lung CSCs.

## Figures and Tables

**Table 1:** List of primers used in the real-time PCR

Gene	Accession	Sense primer (5” - 3”)	Antisense primer (5” - 3”)	Product size (bp)
*ALDH1A1*	NM_000689	CGAGGAACAGTGTGGGTGAA	AGGATAGGACTTGGGGGTCA	378
*RARβ*	NM_000965.4	AGTGCTAAAGGTGCAGAGCG	GTGACTGACTGACCCCACTG	193
*PTGS2*	NM_000963.3	ACTGCTCAACACCGGAATTT	CAAGGGAGTCGGGCAATCAT	290
*CYP24A1*	NM_000782.4	CGCATCTTCCATTTGGCGTT	AATACCACCATCTGAGGCGT	215
*EDN1*	NM_001955.4	GCTGCCTTTTCTCCCCGTTA	AGCGCCTAAGACTGCTGTT	231
*IL1β*	NM_000576.2	GGCTGCTCTGGGATTCTCTT	TGGAGAACACCACTTGTTGC	534
*BIRC5*	NM_00168.2	AGGACCACCGCATCTCTACA	TGTTCCTCTATGGGGTCGTC	187
*GAPDH*	NM_001289746.1	ACACCCACTCCTCCACCTTT	TAGCCAAATTCGTTTGTCATACC	95

**Table 2:** List of Taqman^®^ gene expression primers

Accession number	Gene symbol
Hs01053049_s1	*SOX2*
Hs00999632_g1	*OCT4*
Hs04399610_g1	*NANOG*
Hs00358836_m1	*KLF4*
Hs02758991_g1	*GAPDH*

**Table 3:** Differential expressed genes (DEGs) in various group in microarray data analysis

Sample comparison	Number of identified genes (FC>2.0; *p*<0.05)		
	Total	Up-regulated	Down regulated
G1 normal vs G1 silencing	246	78 (31.7%)	168 (68.3%)
G2 normal vs G2 silencing	411	315 (76.6%)	96 (23.4%)
G3 normal vs G3 silencing	369	175 (47.4%)	194 (52.6%)

*GI - A549 parental; G2 - A549 CD166+EpCAM+; G3 - A549 CD166-EpCAM-.

**Table 4:** Differential expressed genes (DEGs) conserved in all three groups

Gene Symbol	Description	G1	G2	G3
*SESN2*	Sestrin 2	-2.02	2.31	-2.06
*CATSPERB*	Catsper channel auxiliary subunit beta	4.57	-2.04	2.43
*LOC105372955*	Uncharacterised LOC105372955	2.31	3.71	3.32
*CP*	Ceruloplasmin (ferroxidase)	2.34	-2.44	2.02
*NEFL*	Neurofilament, light polypeptide	-2.26	-2.04	-3.26
*IFNE*	Interferon, epsilon	4.62	-2.06	2.98
*TSPAN7*	Tetraspanin 7	-3.85	-2.78	-2.21

*GI - A549 parental; G2 - A549 CD166+EpCAM+; G3 - A549 CD166-EpCAM-.
